# Authors’ Response to Peer Reviews of “Emergence of the First Strains of SARS-CoV-2 Lineage B.1.1.7 in Romania: Genomic Analysis”

**DOI:** 10.2196/32293

**Published:** 2021-08-13

**Authors:** Andrei Lobiuc, Mihai Dimian, Olga Sturdza, Roxana Filip, Mihai Covasa

**Affiliations:** 1 Department of Human Health and Development College of Physical Exercise and Sport Stefan cel Mare University of Suceava Suceava Romania; 2 Department of Computers, Electronics and Automation College of Electrical Engineering and Computer Science Stefan cel Mare University of Suceava Suceava Romania; 3 Integrated Center for Research, Development and Innovation in Advanced Materials, Nanotechnologies, and Distributed Systems for Fabrication and Control Stefan cel Mare University of Suceava Suceava Romania; 4 Suceava County Emergency Hospital Suceava Romania

**Keywords:** infectious disease, COVID-19, strain, virus, Romania, transmission, spread, mutation, impact, case study, genome, sequencing, genetics, epidemiology, variant, virology, lineage

*This is the authors’ response to peer-review reports for “Emergence of the First Strains of SARS-CoV-2 Lineage B.1.1.7 in Romania: Genomic Analysis.”*

## Round 1 Review

### Responses to Reviewers

#### Reviewer 1 [1]

1. The manuscript [2] needs some revision of English. It is generally well prepared, but there are several instances in which it could benefit from a professional revision.

*Response*: Per the reviewer’s [1] comment, we have revised the manuscript throughout with an eye toward improving the English, format, and syntax.

#### Reviewer 2 [3]

1. In the Materials and Methods section, authors mentioned that “Twenty samples, collected from patients in the cities of Cluj, Craiova and Suceava counties from Romania were selected for analysis, including patients with possible contacts with UK infected individuals.” In the Introduction section, the authors also described the first few possible UK variant cases in Romania.

Are these 20 cases sequenced by authors related to those cases mentioned in the Introduction? If not, can authors provide some details about the subjects' past travel history? For example, did they stay in UK for more than 2 weeks before they traveled to Romania? And when were these samples collected? The timeline is important to understand how the disease spread and whether they are the first strains of B.1.1.7 in Romania.

*Response*: We thank the reviewer [3] for his/her comment. The 20 cases sequenced were selected by our laboratory in Suceava as part of the ongoing effort of monitoring SARS-CoV-2 spread in Romania. Among the 20 samples, one—later referred to as EPI_ISL_869241 (Suceava)—was carrying the new UK strain.

The other four samples presented in the Results section were sequenced by other laboratories in the country, so there is no connection with the 20 samples sequenced by us.

Information regarding the travel history of the patients was added, where appropriate. Sample collection dates were added to the table in Multimedia Appendix 1.

2. The authors claimed that “the Romanian strains bearing the particular ORF8 mutations described above clearly originated in the UK, which is also supported by the fact that the patient from Suceava county arrived in Romania from the UK.” I have a similar question about the travel details of the patient as well as the timeline.

*Response*: Information regarding the Romanian patient bearing the ORF8 mutation was added in the Results section.

3. From a public health standpoint, how did the authors deal with the “news” of the new variant? Was there any communication with local officials or support for contact tracing?

*Response*: This information was added to the text. In addition, we mentioned that our laboratory is 1 of 4 (at the moment of writing the paper) that reports weekly genomic data to government agencies. These data are then integrated with epidemiological data to inform public health agencies.

4. In the Discussion section, the authors described that “Many European countries, including Romania, lag in genomic sequencing”. Can the authors provide more details about why Romania lags in genomic sequencing for COVID? For example, cost, equipment, access to labs/institutes. This can help readers and other researchers to understand the issue.

*Response*: The information was added to the text.
